# Machine-Learning-Based Disease Diagnosis and Prediction: Progress, Perspectives, and the Path Forward

**DOI:** 10.3390/diagnostics16121844

**Published:** 2026-06-15

**Authors:** Fakhar Abbas

**Affiliations:** Centre for Trusted Internet & Community, National University of Singapore, Singapore 119077, Singapore; fakhar.5@nus.edu.sg or fakhar.14@hotmail.com

## 1. Introduction

The convergence of machine learning (ML) and clinical medicine has significantly reshaped modern healthcare. Over the past decade, the rapid expansion of health-related data—including electronic health records, medical imaging, genomic data, wearable sensor outputs, and multimodal biosignals—has created both substantial analytical challenges and unprecedented opportunities. While traditional statistical and diagnostic approaches remain foundational, they are often limited in their ability to extract actionable insights from high-dimensional and heterogeneous data at scale. In this context, ML and deep learning (DL) methods have emerged as powerful tools capable of identifying complex, nonlinear patterns and, in certain applications, achieving diagnostic performance comparable to that of experienced clinicians [1,2,3,4].

Despite this progress, several critical challenges remained unresolved at the inception of this Special Issue. These include the generalizability of ML models across diverse populations, the interpretability of black-box systems, the integration of multimodal data, and the limited evaluation of ML approaches across a broad spectrum of diseases.

This Special Issue of *Diagnostics*, entitled “Machine-Learning-Based Disease Diagnosis and Prediction”, was conceived to address these challenges by bringing together original research, review articles, and methodological studies spanning diverse ML-assisted diagnostic applications. Following a rigorous peer-review process, 30 papers were accepted, representing contributions from research groups across Asia, Europe, the Middle East, and Latin America. The breadth of topics—including oncology, neurology, cardiology, nephrology, respiratory medicine, ophthalmology, dentistry, and infectious diseases—highlights both the versatility of ML methodologies and their growing translational relevance. Collectively, these contributions provide a timely snapshot of a rapidly advancing field that continues to face important methodological and clinical barriers.

## 2. Overview of the Published Papers

The thirty papers featured in this Special Issue span multiple clinical domains and methodological approaches. The contributions are summarized below according to thematic clusters.

### 2.1. Medical Imaging and Radiological AI

Medical imaging represents the most prominent domain in this Special Issue, with 11 contributions covering radiography, histopathology, computed tomography (CT), and ophthalmological imaging.

Abd El-Aziz et al. (contribution 1) propose a deep learning fusion model for multiclass classification of lung and colon cancer using histopathological images. By integrating ResNet-101V2, NASNetMobile, and EfficientNet-B0, the model achieved 99.94% accuracy on the LC25000 dataset, demonstrating the effectiveness of feature fusion across complementary architectures.

Abd El-Ghany et al. (contribution 2) introduce FracFusionNet, a multi-level feature fusion convolutional network for bone fracture detection in radiographic images. By combining hierarchical feature representations, the model improved sensitivity across fracture types and offered a computationally efficient approach to automated radiographic screening in emergency and orthopaedic settings.

Yanar et al. (contribution 3) present CELM, an ensemble deep learning model for early cardiomegaly diagnosis in chest radiography. Importantly, the study included external validation across multiple centres, underscoring that strong internal performance alone is insufficient to establish clinical readiness for imaging AI systems.

Hayat et al. (contribution 4) have developed a hybrid CNN–LSTM–Attention architecture for medical image analysis and disease diagnosis. By combining spatial feature extraction, temporal context modelling, and attention mechanisms, the model improved performance across multi-disease image classification tasks.

Likewise, Khan et al. (contribution 5) examine the use of large-scale public datasets for AI-driven chest X-ray analysis and diagnosis. Their findings suggest that training on heterogeneous public data can substantially improve robustness and generalizability, offering a practical strategy for addressing the limitations of single-centre datasets.

Radke et al. (contribution 6) investigate radiomics combined with optimised windowing techniques for detecting dens axis fractures in CT scans. The study showed that preprocessing choices, particularly windowing parameters, can meaningfully influence model performance in challenging skeletal trauma detection tasks.

Bang et al. (contribution 7) have developed a CT-based radiomics model for classifying fat-poor small renal tumour subtypes. Using XGBoost as the optimal classifier, the model achieved an average AU-PRC of 0.757 across five tumour subtypes from single-phase CT, supporting the value of radiomics-based ML in renal oncology.

Similarly, Ficici et al. (contribution 8) introduce a decision-tree-based system for temporal lobe epilepsy focus detection using correlated brain MRI and EEG data. The proposed approach achieved complete concordance with physicians on EEG findings and outperformed physician-only assessment in the combined MRI-EEG task, highlighting the clinical value of multimodal integration.

Nechaev et al. (contribution 9) evaluate the diagnostic accuracy of a multi-target AI service for simultaneous assessment of 16 pathological features on chest and abdominal CT. Their results suggest that integrated AI systems may help streamline radiological workflows and reduce reporting burden.

Faiella et al. (contribution 10) examined AI models for longitudinal CT analysis of epicardial adipose tissue changes following immunotherapy. The study supports the feasibility of AI-assisted serial imaging analysis as a non-invasive tool for monitoring cardiac toxicity during cancer treatment.

Finally, Wu et al. (contribution 11) develop a nomogram for osteoporosis risk stratification using trabecular parameters derived from low-dose CT. Their work supports opportunistic screening from routine CT data as a practical approach to improving osteoporosis detection.

### 2.2. Prediction Using Structured Clinical and Laboratory Data

A substantial cluster of contributions demonstrated the value of ML for risk stratification and outcome prediction using structured clinical, laboratory, and epidemiological data.

Petrović et al. (contribution 12) apply interpretable ML to predict in-hospital mortality after mechanical thrombectomy for anterior circulation large vessel occlusion. Their Pre-MT and Post-MT models achieved AUCs of 0.79 and 0.84, respectively, with baseline NIHSS and age emerging as the most important predictors.

Kapsner et al. (contribution 13) use ML and feature importance analysis to identify mortality risk factors after surgery for congenital heart defects in paediatric patients. Their random survival forest and XGBoost models achieved C-indices of 0.85 and 0.79, with postoperative serum creatinine consistently identified as a key predictor.

Guzmán-Muñoz et al. (contribution 14) a supervised ML model for predicting in-hospital mortality after hip fracture in older adults. Using SHAP-based explainability, the study identified key clinical predictors and demonstrated the value of ML for targeted risk stratification in a high-risk orthopaedic population.

Liu et al. (contribution 15) apply ML to predict the incidence of type 2 diabetes in a 10-year longitudinal cohort from Taiwan. Random forest, logistic regression, and XGBoost all achieved 98–99% accuracy, and free thyroxine emerged as a previously underappreciated predictor.

D’Aversa et al. (contribution 16) integrate host genetics and clinical variables in ML models for predicting COVID-19 prognosis in the FeMiNa Study. Their findings show that combining genetic and clinical data improves prognostic performance over clinical data alone, offering a useful framework for personalised infectious disease prediction.

Iftikhar et al. (contribution 17) apply ensemble ML models to early-stage chronic kidney disease detection using routine laboratory data. The study demonstrated high sensitivity at early disease stages, supporting ML-based screening for earlier referral and intervention.

Yoon et al. (contribution 18) evaluate prediction models incorporating the Hearing Handicap Inventory for the Elderly in patients with chronic otitis media. Logistic regression outperformed random forest, with an AUC of 0.73 and accuracy of 73.56%, highlighting both the utility and current limitations of predictive modelling in otology.

### 2.3. Explainability, Interpretability, and Validation Methodology

Interpretability emerged as a cross-cutting and recurring theme, with several contributions specifically addressing explainable AI frameworks and the methodological rigor required for trustworthy clinical ML.

Aladhadh (contribution 19) developed an explainable ensemble and deep learning framework for Parkinson’s disease detection from voice biomarkers. By combining SHAP analysis with ensemble learning, the study showed that high accuracy and interpretability can be achieved simultaneously.

Omodunbi et al. (contribution 20) apply stacked ensemble learning to Parkinson’s disease classification using telemonitoring vocal features, with particular emphasis on subject-wise validation. The marked drop in performance from conventional cross-validation to subject-wise evaluation clearly illustrates the risk of data leakage in recording-based approaches.

Chang et al. (contribution 21) apply explainable ML models to predict FEV1 in non-smoking Taiwanese men aged 45–55 years. SHAP-based analysis identified key pulmonary predictors within this population, illustrating how XAI can support population-level screening and early intervention.

### 2.4. Oncology Applications

Several contributions extended ML across the cancer care continuum, including diagnosis, prognosis, and molecular classification.

Vazquez et al. (contribution 22) conduct a scoping review of ML and DL applications in cervical cancer diagnosis, prognosis, and treatment. The review found the strongest evidence for image-based diagnostic tasks, while prognostic and treatment applications remain emerging areas of growth.

Likewise, Aydın et al. (contribution 23) address mesothelioma diagnosis using image segmentation and class-based deep feature transformations. Their approach improved classification accuracy for this rare and diagnostically challenging thoracic malignancy.

Finally, Lee et al. (contribution 24) present an ensemble algorithm based on gene selection, data augmentation, and boosting for ovarian cancer classification. Their 10-gene ensemble achieved 98.21% accuracy overall and 100% accuracy for subtype discrimination.

### 2.5. Ophthalmology and Specialised Diagnostics

Two contributions illustrate the application of ML in ophthalmological screening and the emergence of low-cost device-based diagnostic tools.

Khalid et al. (contribution 25) introduce CAD-EYE, an automated multi-eye disease classification system using feature fusion and fluorescence imaging for improved interpretability. Trained on 65,871 fundus images, the model achieved 98% accuracy and outperformed several widely used architectures.

Su et al. (contribution 26) validate a smartphone-based eye-tracking algorithm using the CHT_TM approach. The method reduced execution time substantially while maintaining high accuracy, supporting the feasibility of low-cost eye-movement tracking for scalable screening applications.

### 2.6. Dental and Oral Health Applications

Two contributions extend the reach of ML into structured dental clinical data, addressing endodontic and periodontal treatment outcome prediction.

Bennasar et al. (contribution 27) integrate ML and DL to predict non-surgical root canal treatment outcomes using two-dimensional periapical radiographs. Their combined DL-LR model outperformed clinician prognosis in sensitivity, negative predictive value, and accuracy.

Al-Sharqi et al. (contribution 28) compare unsupervised ML models with logistic regression for predicting nonsurgical periodontal treatment outcomes using tooth-related clinical factors. Their findings support the use of ML for structured dental data and evidence-based treatment planning.

### 2.7. Emerging and System-Level Applications

The final cluster of contributions addresses emerging AI paradigms and broader healthcare system applications, signalling directions that are likely to define the field’s next phase of development.

Khamaysi et al. (contribution 29) review the role of ChatGPT in dermatology diagnostics, exploring its application in natural language processing for clinical data interpretation, differential diagnosis assistance, and patient communication. While the review highlights ChatGPT’s considerable promise for NLP-based diagnostic support, it also identifies the critical limitation of the inability to perform direct image analysis, suggesting that the integration of large language models with vision-based convolutional neural network tools represents a compelling and imminent frontier for comprehensive dermatological AI.

Rani et al. (contribution 30) provide a comprehensive review of ML-powered smart healthcare systems in the era of big data, mapping applications across diagnostic ecosystems while systematically examining challenges and ethical implications. Their analysis identifies federated learning and robust ethical AI governance frameworks as foundational priorities, framing privacy-preserving distributed learning not merely as a technical option but as a structural necessity for the responsible scaling of healthcare AI.

## 3. Future Directions

Several research priorities emerge from the body of work assembled in this Special Issue. First, external validation and prospective clinical evaluation remain the most urgent unmet needs. Many published models were trained and tested on single-centre datasets or public benchmarks, with limited evidence of sustained performance under real-world conditions characterized by population shifts, device variability, and incomplete data. Rigorous multicentre and multinational validation is therefore essential before such systems can be considered ready for routine clinical use [5].

Second, interpretability should be treated as a core design principle rather than a post hoc addition. The studies presented here show that explainable AI methods, particularly SHAP-based analyses, can strengthen clinical insight, identify actionable predictors, and build user confidence when incorporated thoughtfully into model development pipelines. Third, privacy-preserving and federated learning approaches deserve substantially greater emphasis as data-governance requirements tighten globally and cross-institutional collaboration becomes increasingly necessary to train robust, generalizable models.

Fourth, multimodal data integration—combining imaging, genomics, laboratory findings, and patient-reported information—remains one of the most promising and underexplored frontiers for improving diagnostic precision. However, robust methods for handling missing or incomplete modalities in real clinical settings are still needed. Fifth, stronger standardization in benchmarking, reporting, and study design is necessary to reduce the persistent problem of inflated or non-transferable performance claims. Frameworks such as TRIPOD + AI provide important guidance in this regard [5]. Finally, future research should expand further into underrepresented domains, including mental health, autoimmune disease, and other clinically challenging areas where diagnostic uncertainty remains high, and ML may offer genuine benefit.

## 4. Conclusions

The contributions compiled in this Special Issue demonstrate that ML-based disease diagnosis and prediction have evolved from an emerging research area into a credible and increasingly influential paradigm across multiple clinical domains. Spanning medical imaging, clinical risk prediction, oncology, ophthalmology, dentistry, and healthcare systems, the 30 papers collectively reflect both the remarkable breadth of current ML applications in medicine and the persistent methodological challenges that must be addressed before these systems can be fully integrated into clinical practice. This collection underscores that continued progress in the field must be guided by methodological rigor, interpretability, external validation, and sustained attention to fairness and equitable implementation.

The author thanks all contributing authors for their valuable work, the reviewers for their careful and constructive evaluations, and the editorial team at *Diagnostics* for their support throughout the development of this Special Issue. It is hoped that the studies gathered here will stimulate further interdisciplinary research and advance intelligent diagnostic systems that are not only accurate but also trustworthy, equitable, and clinically meaningful.
